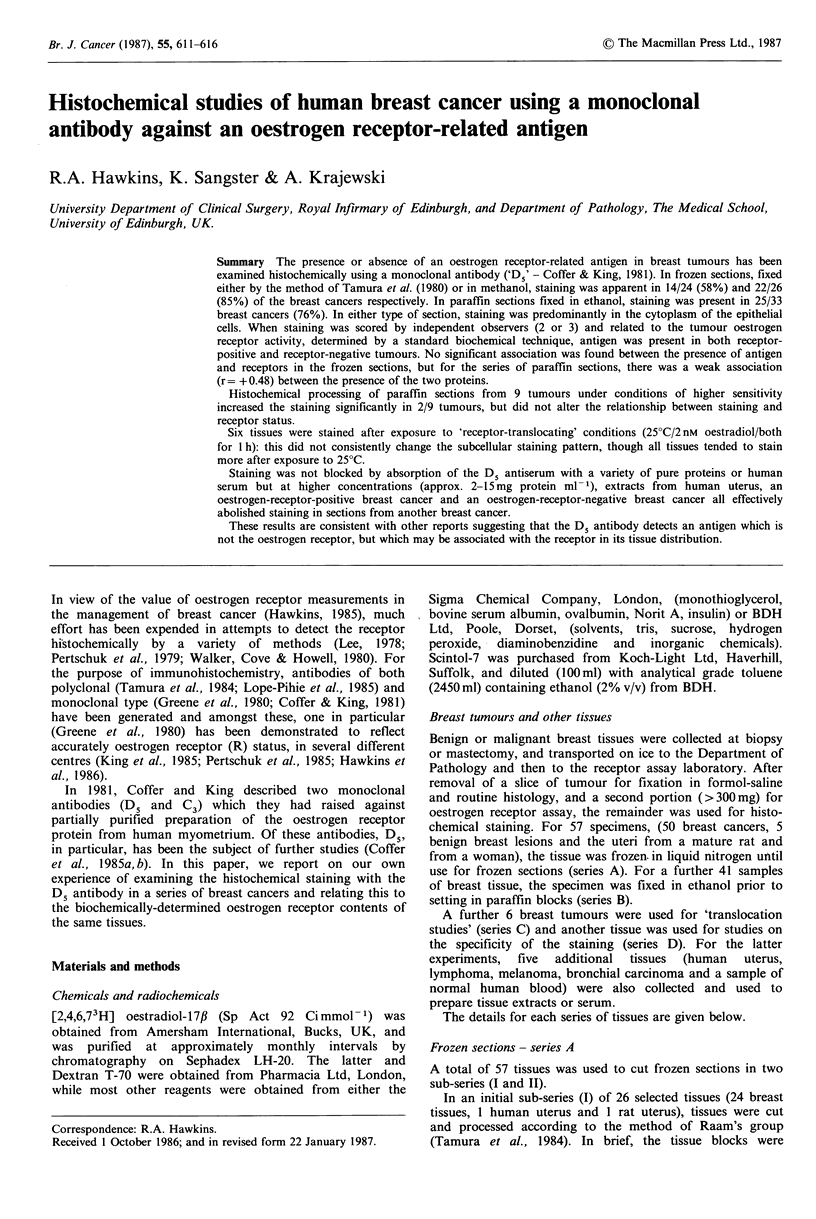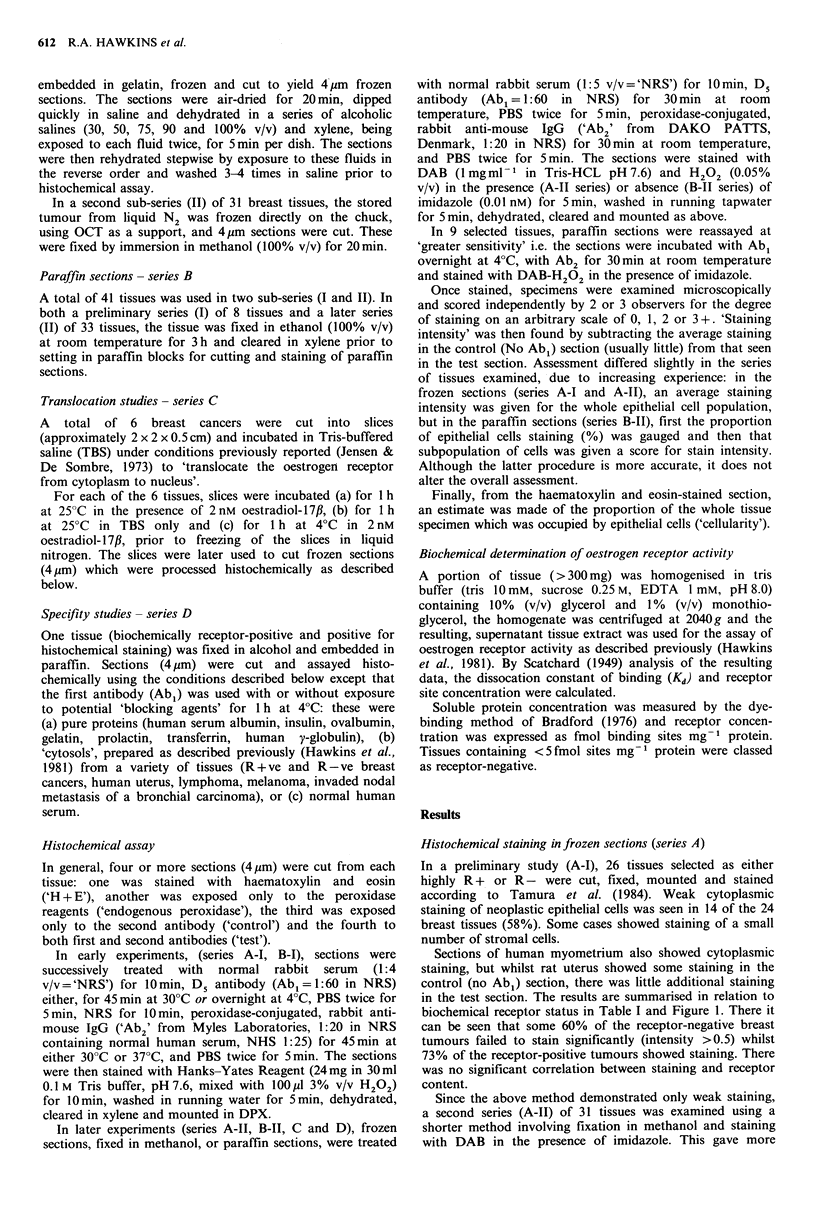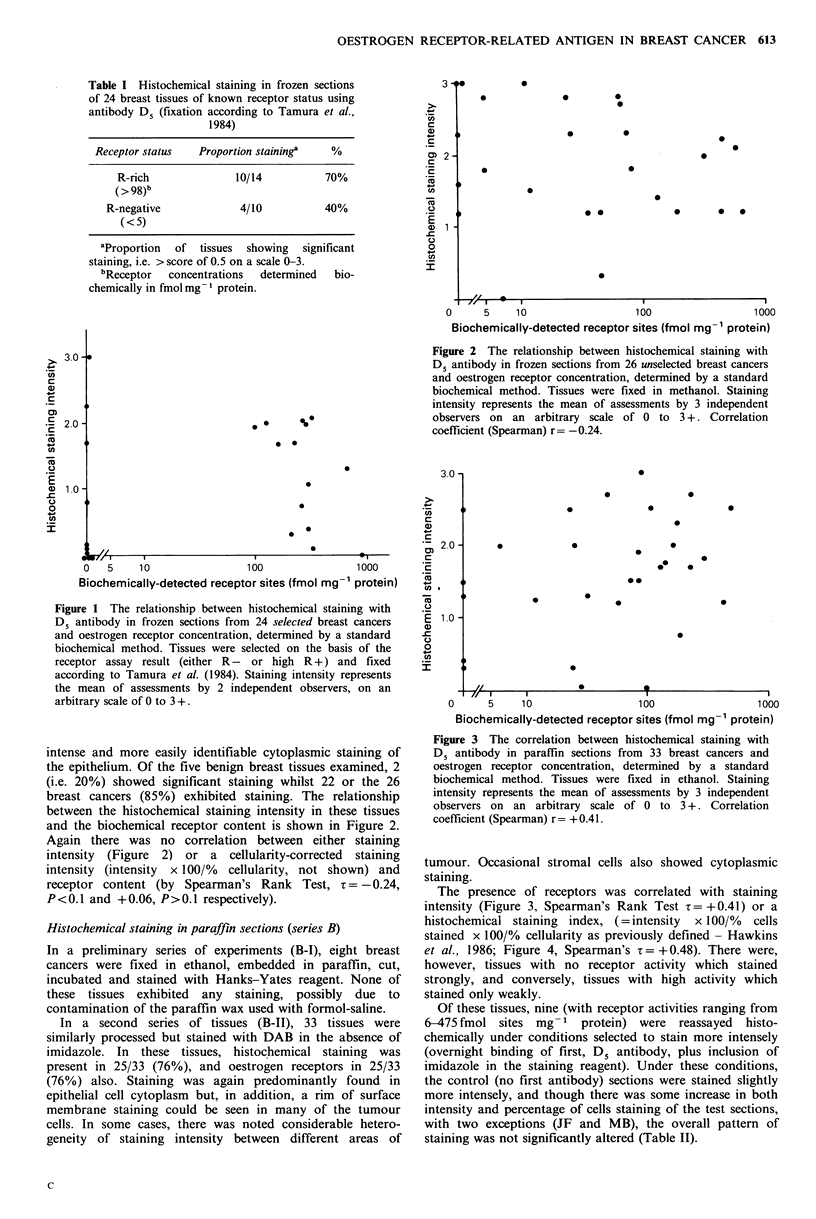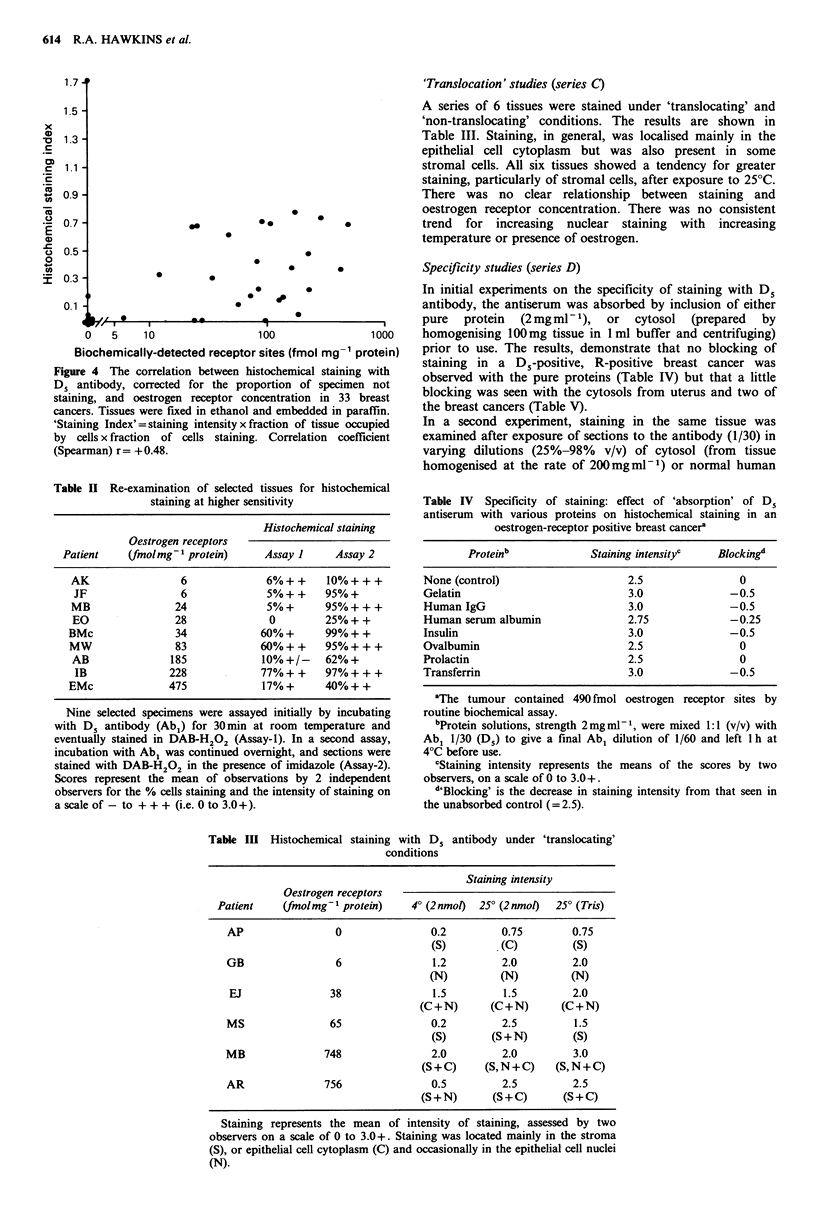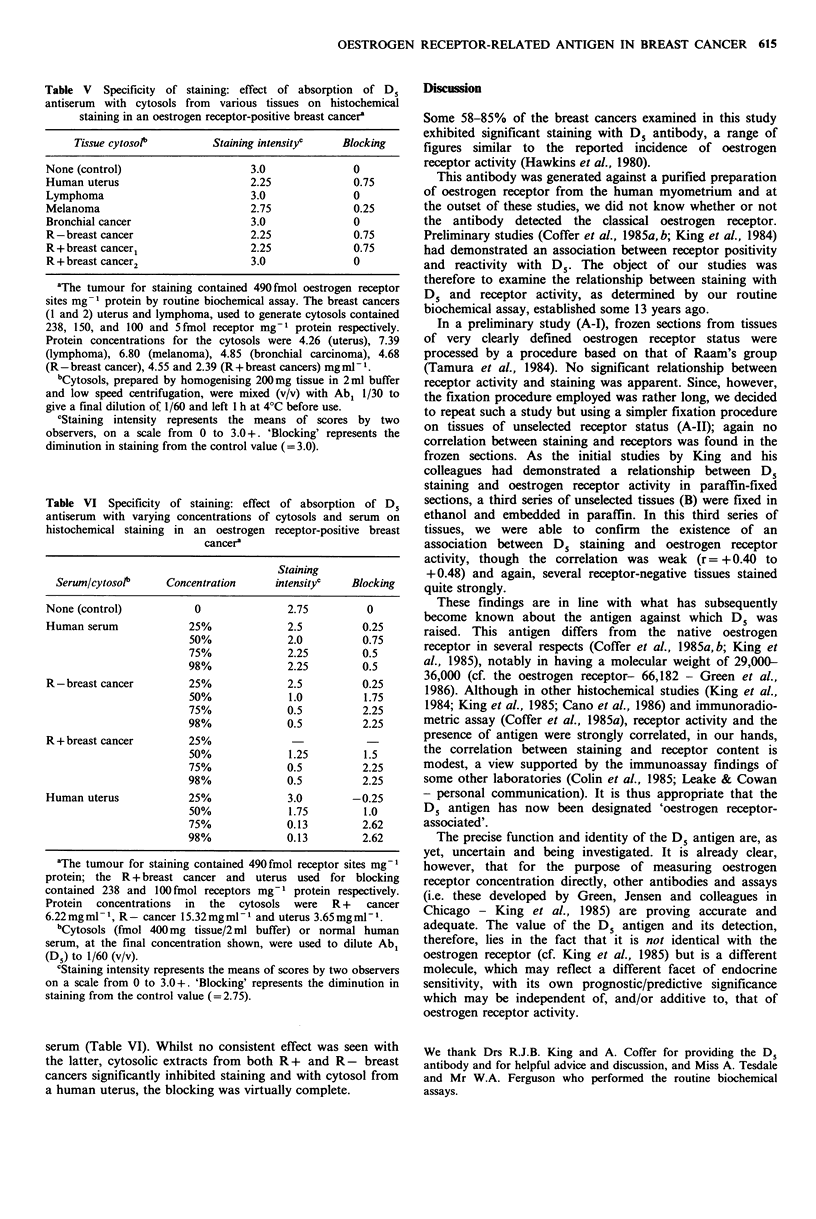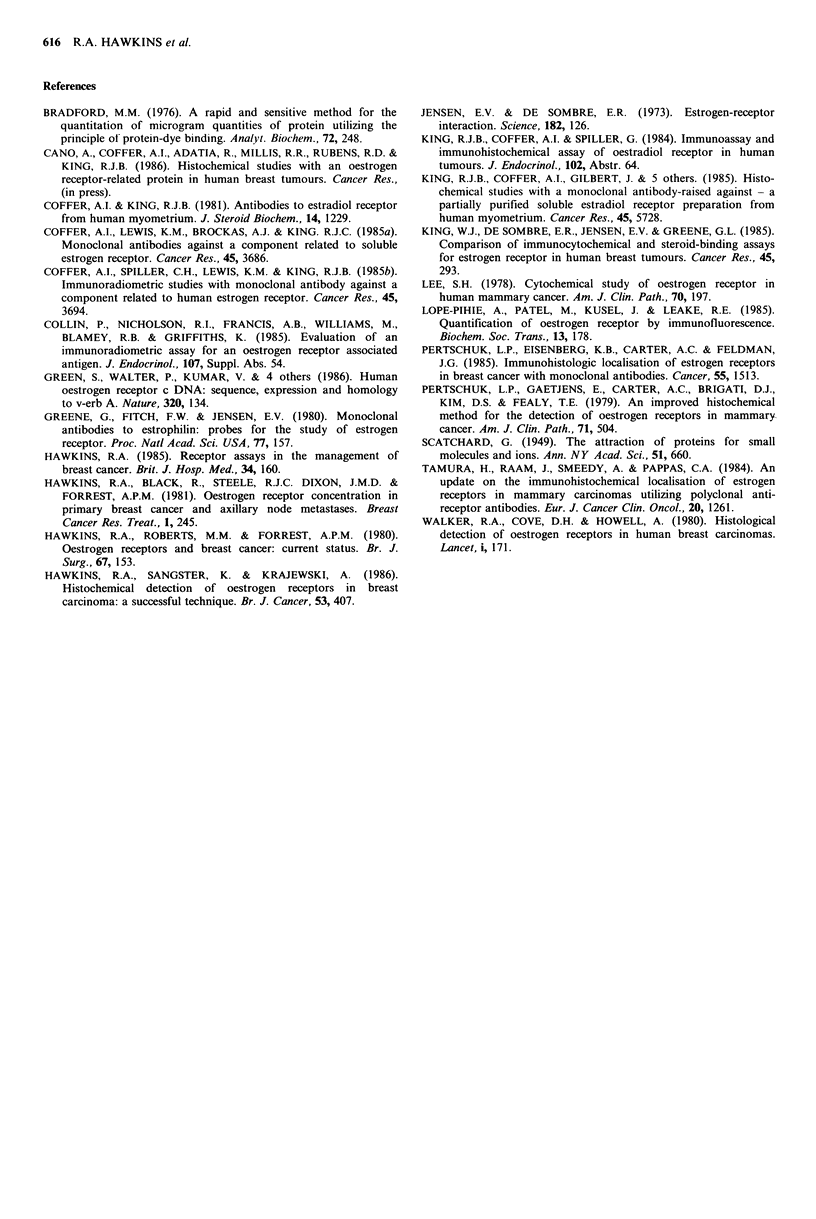# Histochemical studies of human breast cancer using a monoclonal antibody against an oestrogen receptor-related antigen.

**DOI:** 10.1038/bjc.1987.124

**Published:** 1987-06

**Authors:** R. A. Hawkins, K. Sangster, A. Krajewski

## Abstract

The presence or absence of an oestrogen receptor-related antigen in breast tumours has been examined histochemically using a monoclonal antibody ('Ds' - Coffer & King, 1981). In frozen sections, fixed either by the method of Tamura et al. (1980) or in methanol, staining was apparent in 14/24 (58%) and 22/26 (85%) of the breast cancers respectively. In paraffin sections fixed in ethanol, staining was present in 25/33 breast cancers (76%). In either type of section, staining was predominantly in the cytoplasm of the epithelial cells. When staining was scored by independent observers (2 or 3) and related to the tumour oestrogen receptor activity, determined by a standard biochemical technique, antigen was present in both receptor-positive and receptor-negative tumours. No significant association was found between the presence of antigen and receptors in the frozen sections, but for the series of paraffin sections, there was a weak association (r = +0.48) between the presence of the two proteins. Histochemical processing of paraffin sections from 9 tumours under conditions of higher sensitivity increased the staining significantly in 2/9 tumours, but did not alter the relationship between staining and receptor status. Six tissues were stained after exposure to 'receptor-translocating' conditions (25 degrees C/2 nM oestradiol/both for 1 h): this did not consistently change the subcellular staining pattern, though all tissues tended to stain more after exposure to 25 degrees C. Staining was not blocked by absorption of the D5 antiserum with a variety of pure proteins or human serum but at higher concentrations (approx. 2-15 mg protein ml-1), extracts from human uterus, an oestrogen-receptor-positive breast cancer and an oestrogen-receptor-negative breast cancer all effectively abolished staining in sections from another breast cancer. These results are consistent with other reports suggesting that the D5 antibody detects an antigen which is not the oestrogen receptor, but which may be associated with the receptor in its tissue distribution.


*Br. J. Cancer* (1987), **55**, 611–616 © The Macmillan Press Ltd., 1987

### Histochemical studies of human breast cancer using a monoclonal antibody against an oestrogen receptor-related antigen

R.A. Hawkins, K. Sangster & A. Krajewski

*University Department of Clinical Surgery, Royal Infirmary of Edinburgh, and Department of Pathology, The Medical School, University of Edinburgh, UK.*

**Summary** The presence or absence of an oestrogen receptor-related antigen in breast tumours has been examined histochemically using a monoclonal antibody ('$D_5$' – Coffer & King, 1981). In frozen sections, fixed either by the method of Tamura *et al.* (1980) or in methanol, staining was apparent in 14/24 (58%) and 22/26 (85%) of the breast cancers respectively. In paraffin sections fixed in ethanol, staining was present in 25/33 breast cancers (76%). In either type of section, staining was predominantly in the cytoplasm of the epithelial cells. When staining was scored by independent observers (2 or 3) and related to the tumour oestrogen receptor activity, determined by a standard biochemical technique, antigen was present in both receptor-positive and receptor-negative tumours. No significant association was found between the presence of antigen and receptors in the frozen sections, but for the series of paraffin sections, there was a weak association ($r = +0.48$) between the presence of the two proteins.

Histochemical processing of paraffin sections from 9 tumours under conditions of higher sensitivity increased the staining significantly in 2/9 tumours, but did not alter the relationship between staining and receptor status.

Six tissues were stained after exposure to 'receptor-translocating' conditions (25°C/2 nM oestradiol/both for 1 h): this did not consistently change the subcellular staining pattern, though all tissues tended to stain more after exposure to 25°C.

Staining was not blocked by absorption of the $D_5$ antiserum with a variety of pure proteins or human serum but at higher concentrations (approx. 2–15 mg protein $ml^{-1}$), extracts from human uterus, an oestrogen-receptor-positive breast cancer and an oestrogen-receptor-negative breast cancer all effectively abolished staining in sections from another breast cancer.

These results are consistent with other reports suggesting that the $D_5$ antibody detects an antigen which is not the oestrogen receptor, but which may be associated with the receptor in its tissue distribution.

In view of the value of oestrogen receptor measurements in the management of breast cancer (Hawkins, 1985), much effort has been expended in attempts to detect the receptor histochemically by a variety of methods (Lee, 1978; Pertschuk *et al.*, 1979; Walker, Cove & Howell, 1980). For the purpose of immunohistochemistry, antibodies of both polyclonal (Tamura *et al.*, 1984; Lope-Pihie *et al.*, 1985) and monoclonal type (Greene *et al.*, 1980; Coffer & King, 1981) have been generated and amongst these, one in particular (Greene *et al.*, 1980) has been demonstrated to reflect accurately oestrogen receptor (R) status, in several different centres (King *et al.*, 1985; Pertschuk *et al.*, 1985; Hawkins *et al.*, 1986).

In 1981, Coffer and King described two monoclonal antibodies ($D_5$ and $C_3$) which they had raised against partially purified preparation of the oestrogen receptor protein from human myometrium. Of these antibodies, $D_5$, in particular, has been the subject of further studies (Coffer *et al.*, 1985*a*,*b*). In this paper, we report on our own experience of examining the histochemical staining with the $D_5$ antibody in a series of breast cancers and relating this to the biochemically-determined oestrogen receptor contents of the same tissues.

### Materials and methods

### *Chemicals and radiochemicals*

[2,4,6,7$^3$H] oestradiol-17$\beta$ (Sp Act 92 Ci mmol$^{-1}$) was obtained from Amersham International, Bucks, UK, and was purified at approximately monthly intervals by chromatography on Sephadex LH-20. The latter and Dextran T-70 were obtained from Pharmacia Ltd, London, while most other reagents were obtained from either the Sigma Chemical Company, London, (monothioglycerol, bovine serum albumin, ovalbumin, Norit A, insulin) or BDH Ltd, Poole, Dorset, (solvents, tris, sucrose, hydrogen peroxide, diaminobenzidine and inorganic chemicals). Scintol-7 was purchased from Koch-Light Ltd, Haverhill, Suffolk, and diluted (100 ml) with analytical grade toluene (2450 ml) containing ethanol (2% v/v) from BDH.

### *Breast tumours and other tissues*

Benign or malignant breast tissues were collected at biopsy or mastectomy, and transported on ice to the Department of Pathology and then to the receptor assay laboratory. After removal of a slice of tumour for fixation in formol-saline and routine histology, and a second portion (>300 mg) for oestrogen receptor assay, the remainder was used for histochemical staining. For 57 specimens, (50 breast cancers, 5 benign breast lesions and the uteri from a mature rat and from a woman), the tissue was frozen in liquid nitrogen until use for frozen sections (series A). For a further 41 samples of breast tissue, the specimen was fixed in ethanol prior to setting in paraffin blocks (series B).

A further 6 breast tumours were used for 'translocation studies' (series C) and another tissue was used for studies on the specificity of the staining (series D). For the latter experiments, five additional tissues (human uterus, lymphoma, melanoma, bronchial carcinoma and a sample of normal human blood) were also collected and used to prepare tissue extracts or serum.

The details for each series of tissues are given below.

### *Frozen sections – series A*

A total of 57 tissues was used to cut frozen sections in two sub-series (I and II).

In an initial sub-series (I) of 26 selected tissues (24 breast tissues, 1 human uterus and 1 rat uterus), tissues were cut and processed according to the method of Raam's group (Tamura *et al.*, 1984). In brief, the tissue blocks were

Correspondence: R.A. Hawkins.
Received 1 October 1986; and in revised form 22 January 1987.

embedded in gelatin, frozen and cut to yield 4 μm frozen sections. The sections were air-dried for 20 min, dipped quickly in saline and dehydrated in a series of alcoholic salines (30, 50, 75, 90 and 100% v/v) and xylene, being exposed to each fluid twice, for 5 min per dish. The sections were then rehydrated stepwise by exposure to these fluids in the reverse order and washed 3–4 times in saline prior to histochemical assay.

In a second sub-series (II) of 31 breast tissues, the stored tumour from liquid $N_2$ was frozen directly on the chuck, using OCT as a support, and 4 μm sections were cut. These were fixed by immersion in methanol (100% v/v) for 20 min.

*Paraffin sections – series B*

A total of 41 tissues was used in two sub-series (I and II). In both a preliminary series (I) of 8 tissues and a later series (II) of 33 tissues, the tissue was fixed in ethanol (100% v/v) at room temperature for 3 h and cleared in xylene prior to setting in paraffin blocks for cutting and staining of paraffin sections.

*Translocation studies – series C*

A total of 6 breast cancers were cut into slices (approximately 2 × 2 × 0.5 cm) and incubated in Tris-buffered saline (TBS) under conditions previously reported (Jensen & De Sombre, 1973) to 'translocate the oestrogen receptor from cytoplasm to nucleus'.

For each of the 6 tissues, slices were incubated (a) for 1 h at 25°C in the presence of 2 nM oestradiol-17β, (b) for 1 h at 25°C in TBS only and (c) for 1 h at 4°C in 2 nM oestradiol-17β, prior to freezing of the slices in liquid nitrogen. The slices were later used to cut frozen sections (4 μm) which were processed histochemically as described below.

*Specifity studies – series D*

One tissue (biochemically receptor-positive and positive for histochemical staining) was fixed in alcohol and embedded in paraffin. Sections (4 μm) were cut and assayed histochemically using the conditions described below except that the first antibody ($Ab_1$) was used with or without exposure to potential 'blocking agents' for 1 h at 4°C: these were (a) pure proteins (human serum albumin, insulin, ovalbumin, gelatin, prolactin, transferrin, human γ-globulin), (b) 'cytosols', prepared as described previously (Hawkins *et al.*, 1981) from a variety of tissues (R+ve and R−ve breast cancers, human uterus, lymphoma, melanoma, invaded nodal metastasis of a bronchial carcinoma), or (c) normal human serum.

*Histochemical assay*

In general, four or more sections (4 μm) were cut from each tissue: one was stained with haematoxylin and eosin ('H+E'), another was exposed only to the peroxidase reagents ('endogenous peroxidase'), the third was exposed only to the second antibody ('control') and the fourth to both first and second antibodies ('test').

In early experiments, (series A-I, B-I), sections were successively treated with normal rabbit serum (1:4 v/v = 'NRS') for 10 min, $D_5$ antibody ($Ab_1$ = 1:60 in NRS) either, for 45 min at 30°C *or* overnight at 4°C, PBS twice for 5 min, NRS for 10 min, peroxidase-conjugated, rabbit anti-mouse IgG ('$Ab_2$' from Myles Laboratories, 1:20 in NRS containing normal human serum, NHS 1:25) for 45 min at either 30°C or 37°C, and PBS twice for 5 min. The sections were then stained with Hanks–Yates Reagent (24 mg in 30 ml 0.1 M Tris buffer, pH 7.6, mixed with 100 μl 3% v/v $H_2O_2$) for 10 min, washed in running water for 5 min, dehydrated, cleared in xylene and mounted in DPX.

In later experiments (series A-II, B-II, C and D), frozen sections, fixed in methanol, or paraffin sections, were treated with normal rabbit serum (1:5 v/v = 'NRS') for 10 min, $D_5$ antibody ($Ab_1$ = 1:60 in NRS) for 30 min at room temperature, PBS twice for 5 min, peroxidase-conjugated, rabbit anti-mouse IgG ('$Ab_2$' from DAKO PATTS, Denmark, 1:20 in NRS) for 30 min at room temperature, and PBS twice for 5 min. The sections were stained with DAB (1 mg ml$^{-1}$ in Tris-HCL pH 7.6) and $H_2O_2$ (0.05% v/v) in the presence (A-II series) or absence (B-II series) of imidazole (0.01 nM) for 5 min, washed in running tapwater for 5 min, dehydrated, cleared and mounted as above.

In 9 selected tissues, paraffin sections were reassayed at 'greater sensitivity' i.e. the sections were incubated with $Ab_1$ overnight at 4°C, with $Ab_2$ for 30 min at room temperature and stained with DAB-$H_2O_2$ in the presence of imidazole.

Once stained, specimens were examined microscopically and scored independently by 2 or 3 observers for the degree of staining on an arbitrary scale of 0, 1, 2 or 3+. 'Staining intensity' was then found by subtracting the average staining in the control (No $Ab_1$) section (usually little) from that seen in the test section. Assessment differed slightly in the series of tissues examined, due to increasing experience: in the frozen sections (series A-I and A-II), an average staining intensity was given for the whole epithelial cell population, but in the paraffin sections (series B-II), first the proportion of epithelial cells staining (%) was gauged and then that subpopulation of cells was given a score for stain intensity. Although the latter procedure is more accurate, it does not alter the overall assessment.

Finally, from the haematoxylin and eosin-stained section, an estimate was made of the proportion of the whole tissue specimen which was occupied by epithelial cells ('cellularity').

*Biochemical determination of oestrogen receptor activity*

A portion of tissue (>300 mg) was homogenised in tris buffer (tris 10 mM, sucrose 0.25 M, EDTA 1 mM, pH 8.0) containing 10% (v/v) glycerol and 1% (v/v) monothioglycerol, the homogenate was centrifuged at 2040 *g* and the resulting, supernatant tissue extract was used for the assay of oestrogen receptor activity as described previously (Hawkins *et al.*, 1981). By Scatchard (1949) analysis of the resulting data, the dissocation constant of binding ($K_d$) and receptor site concentration were calculated.

Soluble protein concentration was measured by the dye-binding method of Bradford (1976) and receptor concentration was expressed as fmol binding sites mg$^{-1}$ protein. Tissues containing <5 fmol sites mg$^{-1}$ protein were classed as receptor-negative.

### Results

*Histochemical staining in frozen sections (series A)*

In a preliminary study (A-I), 26 tissues selected as either highly R+ or R− were cut, fixed, mounted and stained according to Tamura *et al.* (1984). Weak cytoplasmic staining of neoplastic epithelial cells was seen in 14 of the 24 breast tissues (58%). Some cases showed staining of a small number of stromal cells.

Sections of human myometrium also showed cytoplasmic staining, but whilst rat uterus showed some staining in the control (no $Ab_1$) section, there was little additional staining in the test section. The results are summarised in relation to biochemical receptor status in Table I and Figure 1. There it can be seen that some 60% of the receptor-negative breast tumours failed to stain significantly (intensity >0.5) whilst 73% of the receptor-positive tumours showed staining. There was no significant correlation between staining and receptor content.

Since the above method demonstrated only weak staining, a second series (A-II) of 31 tissues was examined using a shorter method involving fixation in methanol and staining with DAB in the presence of imidazole. This gave more

**Table I** Histochemical staining in frozen sections of 24 breast tissues of known receptor status using antibody $D_5$ (fixation according to Tamura *et al.*, 1984)

| *Receptor status* | *Proportion staining*[a] | % |
|---|---|---|
| R-rich (>98)[b] | 10/14 | 70% |
| R-negative (<5) | 4/10 | 40% |

[a]Proportion of tissues showing significant staining, i.e. > score of 0.5 on a scale 0–3.
[b]Receptor concentrations determined biochemically in fmol mg$^{-1}$ protein.

**Figure 1** The relationship between histochemical staining with $D_5$ antibody in frozen sections from 24 *selected* breast cancers and oestrogen receptor concentration, determined by a standard biochemical method. Tissues were selected on the basis of the receptor assay result (either R− or high R+) and fixed according to Tamura *et al.* (1984). Staining intensity represents the mean of assessments by 2 independent observers, on an arbitrary scale of 0 to 3+.

intense and more easily identifiable cytoplasmic staining of the epithelium. Of the five benign breast tissues examined, 2 (i.e. 20%) showed significant staining whilst 22 or the 26 breast cancers (85%) exhibited staining. The relationship between the histochemical staining intensity in these tissues and the biochemical receptor content is shown in Figure 2. Again there was no correlation between either staining intensity (Figure 2) or a cellularity-corrected staining intensity (intensity × 100/% cellularity, not shown) and receptor content (by Spearman's Rank Test, $\tau = -0.24$, $P<0.1$ and $+0.06$, $P>0.1$ respectively).

*Histochemical staining in paraffin sections (series B)*

In a preliminary series of experiments (B-I), eight breast cancers were fixed in ethanol, embedded in paraffin, cut, incubated and stained with Hanks–Yates reagent. None of these tissues exhibited any staining, possibly due to contamination of the paraffin wax used with formol-saline.

In a second series of tissues (B-II), 33 tissues were similarly processed but stained with DAB in the absence of imidazole. In these tissues, histochemical staining was present in 25/33 (76%), and oestrogen receptors in 25/33 (76%) also. Staining was again predominantly found in epithelial cell cytoplasm but, in addition, a rim of surface membrane staining could be seen in many of the tumour cells. In some cases, there was noted considerable heterogeneity of staining intensity between different areas of

**Figure 2** The relationship between histochemical staining with $D_5$ antibody in frozen sections from 26 *un*selected breast cancers and oestrogen receptor concentration, determined by a standard biochemical method. Tissues were fixed in methanol. Staining intensity represents the mean of assessments by 3 independent observers on an arbitrary scale of 0 to 3+. Correlation coefficient (Spearman) r = −0.24.

**Figure 3** The correlation between histochemical staining with $D_5$ antibody in paraffin sections from 33 breast cancers and oestrogen receptor concentration, determined by a standard biochemical method. Tissues were fixed in ethanol. Staining intensity represents the mean of assessments by 3 independent observers on an arbitrary scale of 0 to 3+. Correlation coefficient (Spearman) r = +0.41.

tumour. Occasional stromal cells also showed cytoplasmic staining.

The presence of receptors was correlated with staining intensity (Figure 3, Spearman's Rank Test $\tau = +0.41$) or a histochemical staining index, (= intensity × 100/% cells stained × 100/% cellularity as previously defined – Hawkins *et al.*, 1986; Figure 4, Spearman's $\tau = +0.48$). There were, however, tissues with no receptor activity which stained strongly, and conversely, tissues with high activity which stained only weakly.

Of these tissues, nine (with receptor activities ranging from 6–475 fmol sites mg$^{-1}$ protein) were reassayed histochemically under conditions selected to stain more intensely (overnight binding of first, $D_5$ antibody, plus inclusion of imidazole in the staining reagent). Under these conditions, the control (no first antibody) sections were stained slightly more intensely, and though there was some increase in both intensity and percentage of cells staining of the test sections, with two exceptions (JF and MB), the overall pattern of staining was not significantly altered (Table II).

**Figure 4** The correlation between histochemical staining with $D_5$ antibody, corrected for the proportion of specimen not staining, and oestrogen receptor concentration in 33 breast cancers. Tissues were fixed in ethanol and embedded in paraffin. 'Staining Index' = staining intensity × fraction of tissue occupied by cells × fraction of cells staining. Correlation coefficient (Spearman) r = +0.48.

**Table II** Re-examination of selected tissues for histochemical staining at higher sensitivity

| Patient | Oestrogen receptors (fmol mg⁻¹ protein) | Histochemical staining Assay 1 | Histochemical staining Assay 2 |
|---|---|---|---|
| AK | 6 | 6% + + | 10% + + + |
| JF | 6 | 5% + + | 95% + |
| MB | 24 | 5% + | 95% + + + |
| EO | 28 | 0 | 25% + + |
| BMc | 34 | 60% + | 99% + + |
| MW | 83 | 60% + + | 95% + + + |
| AB | 185 | 10% + / − | 62% + |
| IB | 228 | 77% + + | 97% + + + |
| EMc | 475 | 17% + | 40% + + |

Nine selected specimens were assayed initially by incubating with $D_5$ antibody ($Ab_1$) for 30 min at room temperature and eventually stained in DAB-$H_2O_2$ (Assay-1). In a second assay, incubation with $Ab_1$ was continued overnight, and sections were stained with DAB-$H_2O_2$ in the presence of imidazole (Assay-2). Scores represent the mean of observations by 2 independent observers for the % cells staining and the intensity of staining on a scale of − to + + + (i.e. 0 to 3.0+).

### 'Translocation' studies (series C)

A series of 6 tissues were stained under 'translocating' and 'non-translocating' conditions. The results are shown in Table III. Staining, in general, was localised mainly in the epithelial cell cytoplasm but was also present in some stromal cells. All six tissues showed a tendency for greater staining, particularly of stromal cells, after exposure to 25°C. There was no clear relationship between staining and oestrogen receptor concentration. There was no consistent trend for increasing nuclear staining with increasing temperature or presence of oestrogen.

### Specificity studies (series D)

In initial experiments on the specificity of staining with $D_5$ antibody, the antiserum was absorbed by inclusion of either pure protein ($2\,mg\,ml^{-1}$), or cytosol (prepared by homogenising 100 mg tissue in 1 ml buffer and centrifuging) prior to use. The results, demonstrate that no blocking of staining in a $D_5$-positive, R-positive breast cancer was observed with the pure proteins (Table IV) but that a little blocking was seen with the cytosols from uterus and two of the breast cancers (Table V).

In a second experiment, staining in the same tissue was examined after exposure of sections to the antibody (1/30) in varying dilutions (25%–98% v/v) of cytosol (from tissue homogenised at the rate of $200\,mg\,ml^{-1}$) or normal human

**Table IV** Specificity of staining: effect of 'absorption' of $D_5$ antiserum with various proteins on histochemical staining in an oestrogen-receptor positive breast cancer[a]

| Protein[b] | Staining intensity[c] | Blocking[d] |
|---|---|---|
| None (control) | 2.5 | 0 |
| Gelatin | 3.0 | −0.5 |
| Human IgG | 3.0 | −0.5 |
| Human serum albumin | 2.75 | −0.25 |
| Insulin | 3.0 | −0.5 |
| Ovalbumin | 2.5 | 0 |
| Prolactin | 2.5 | 0 |
| Transferrin | 3.0 | −0.5 |

[a]The tumour contained 490 fmol oestrogen receptor sites by routine biochemical assay.
[b]Protein solutions, strength $2\,mg\,ml^{-1}$, were mixed 1:1 (v/v) with $Ab_1$ 1/30 ($D_5$) to give a final $Ab_1$ dilution of 1/60 and left 1 h at 4°C before use.
[c]Staining intensity represents the means of the scores by two observers, on a scale of 0 to 3.0+.
[d]'Blocking' is the decrease in staining intensity from that seen in the unabsorbed control (=2.5).

**Table III** Histochemical staining with $D_5$ antibody under 'translocating' conditions

| Patient | Oestrogen receptors (fmol mg⁻¹ protein) | Staining intensity 4° (2 nmol) | Staining intensity 25° (2 nmol) | Staining intensity 25° (Tris) |
|---|---|---|---|---|
| AP | 0 | 0.2 (S) | 0.75 (C) | 0.75 (S) |
| GB | 6 | 1.2 (N) | 2.0 (N) | 2.0 (N) |
| EJ | 38 | 1.5 (C+N) | 1.5 (C+N) | 2.0 (C+N) |
| MS | 65 | 0.2 (S) | 2.5 (S+N) | 1.5 (S) |
| MB | 748 | 2.0 (S+C) | 2.0 (S, N+C) | 3.0 (S, N+C) |
| AR | 756 | 0.5 (S+N) | 2.5 (S+C) | 2.5 (S+C) |

Staining represents the mean of intensity of staining, assessed by two observers on a scale of 0 to 3.0+. Staining was located mainly in the stroma (S), or epithelial cell cytoplasm (C) and occasionally in the epithelial cell nuclei (N).

**Table V** Specificity of staining: effect of absorption of $D_5$ antiserum with cytosols from various tissues on histochemical staining in an oestrogen receptor-positive breast cancer[a]

| *Tissue cytosol*[b] | *Staining intensity*[c] | *Blocking* |
|---|---|---|
| None (control) | 3.0 | 0 |
| Human uterus | 2.25 | 0.75 |
| Lymphoma | 3.0 | 0 |
| Melanoma | 2.75 | 0.25 |
| Bronchial cancer | 3.0 | 0 |
| R – breast cancer | 2.25 | 0.75 |
| R + breast cancer$_1$ | 2.25 | 0.75 |
| R + breast cancer$_2$ | 3.0 | 0 |

[a]The tumour for staining contained 490 fmol oestrogen receptor sites $mg^{-1}$ protein by routine biochemical assay. The breast cancers (1 and 2) uterus and lymphoma, used to generate cytosols contained 238, 150, and 100 and 5 fmol receptor $mg^{-1}$ protein respectively. Protein concentrations for the cytosols were 4.26 (uterus), 7.39 (lymphoma), 6.80 (melanoma), 4.85 (bronchial carcinoma), 4.68 (R – breast cancer), 4.55 and 2.39 (R + breast cancers) mg $ml^{-1}$.

[b]Cytosols, prepared by homogenising 200 mg tissue in 2 ml buffer and low speed centrifugation, were mixed (v/v) with $Ab_1$ 1/30 to give a final dilution of 1/60 and left 1 h at 4°C before use.

[c]Staining intensity represents the means of scores by two observers, on a scale from 0 to 3.0+. 'Blocking' represents the diminution in staining from the control value (= 3.0).

**Table VI** Specificity of staining: effect of absorption of $D_5$ antiserum with varying concentrations of cytosols and serum on histochemical staining in an oestrogen receptor-positive breast cancer[a]

| *Serum/cytosol*[b] | *Concentration* | *Staining intensity*[c] | *Blocking* |
|---|---|---|---|
| None (control) | 0 | 2.75 | 0 |
| Human serum | 25% | 2.5 | 0.25 |
| | 50% | 2.0 | 0.75 |
| | 75% | 2.25 | 0.5 |
| | 98% | 2.25 | 0.5 |
| R – breast cancer | 25% | 2.5 | 0.25 |
| | 50% | 1.0 | 1.75 |
| | 75% | 0.5 | 2.25 |
| | 98% | 0.5 | 2.25 |
| R + breast cancer | 25% | — | — |
| | 50% | 1.25 | 1.5 |
| | 75% | 0.5 | 2.25 |
| | 98% | 0.5 | 2.25 |
| Human uterus | 25% | 3.0 | –0.25 |
| | 50% | 1.75 | 1.0 |
| | 75% | 0.13 | 2.62 |
| | 98% | 0.13 | 2.62 |

[a]The tumour for staining contained 490 fmol receptor sites $mg^{-1}$ protein; the R + breast cancer and uterus used for blocking contained 238 and 100 fmol receptors $mg^{-1}$ protein respectively. Protein concentrations in the cytosols were R + cancer 6.22 mg $ml^{-1}$, R – cancer 15.32 mg $ml^{-1}$ and uterus 3.65 mg $ml^{-1}$.

[b]Cytosols (fmol 400 mg tissue/2 ml buffer) or normal human serum, at the final concentration shown, were used to dilute $Ab_1$ ($D_5$) to 1/60 (v/v).

[c]Staining intensity represents the means of scores by two observers on a scale from 0 to 3.0+. 'Blocking' represents the diminution in staining from the control value (= 2.75).

serum (Table VI). Whilst no consistent effect was seen with the latter, cytosolic extracts from both R+ and R– breast cancers significantly inhibited staining and with cytosol from a human uterus, the blocking was virtually complete.

### Discussion

Some 58–85% of the breast cancers examined in this study exhibited significant staining with $D_5$ antibody, a range of figures similar to the reported incidence of oestrogen receptor activity (Hawkins *et al.*, 1980).

This antibody was generated against a purified preparation of oestrogen receptor from the human myometrium and at the outset of these studies, we did not know whether or not the antibody detected the classical oestrogen receptor. Preliminary studies (Coffer *et al.*, 1985*a*,*b*; King *et al.*, 1984) had demonstrated an association between receptor positivity and reactivity with $D_5$. The object of our studies was therefore to examine the relationship between staining with $D_5$ and receptor activity, as determined by our routine biochemical assay, established some 13 years ago.

In a preliminary study (A-I), frozen sections from tissues of very clearly defined oestrogen receptor status were processed by a procedure based on that of Raam's group (Tamura *et al.*, 1984). No significant relationship between receptor activity and staining was apparent. Since, however, the fixation procedure employed was rather long, we decided to repeat such a study but using a simpler fixation procedure on tissues of unselected receptor status (A-II); again no correlation between staining and receptors was found in the frozen sections. As the initial studies by King and his colleagues had demonstrated a relationship between $D_5$ staining and oestrogen receptor activity in paraffin-fixed sections, a third series of unselected tissues (B) were fixed in ethanol and embedded in paraffin. In this third series of tissues, we were able to confirm the existence of an association between $D_5$ staining and oestrogen receptor activity, though the correlation was weak ($r = +0.40$ to $+0.48$) and again, several receptor-negative tissues stained quite strongly.

These findings are in line with what has subsequently become known about the antigen against which $D_5$ was raised. This antigen differs from the native oestrogen receptor in several respects (Coffer *et al.*, 1985*a*,*b*; King *et al.*, 1985), notably in having a molecular weight of 29,000–36,000 (cf. the oestrogen receptor– 66,182 – Green *et al.*, 1986). Although in other histochemical studies (King *et al.*, 1984; King *et al.*, 1985; Cano *et al.*, 1986) and immunoradiometric assay (Coffer *et al.*, 1985*a*), receptor activity and the presence of antigen were strongly correlated, in our hands, the correlation between staining and receptor content is modest, a view supported by the immunoassay findings of some other laboratories (Colin *et al.*, 1985; Leake & Cowan – personal communication). It is thus appropriate that the $D_5$ antigen has now been designated 'oestrogen receptor-associated'.

The precise function and identity of the $D_5$ antigen are, as yet, uncertain and being investigated. It is already clear, however, that for the purpose of measuring oestrogen receptor concentration directly, other antibodies and assays (i.e. these developed by Green, Jensen and colleagues in Chicago – King *et al.*, 1985) are proving accurate and adequate. The value of the $D_5$ antigen and its detection, therefore, lies in the fact that it is *not* identical with the oestrogen receptor (cf. King *et al.*, 1985) but is a different molecule, which may reflect a different facet of endocrine sensitivity, with its own prognostic/predictive significance which may be independent of, and/or additive to, that of oestrogen receptor activity.

We thank Drs R.J.B. King and A. Coffer for providing the $D_5$ antibody and for helpful advice and discussion, and Miss A. Tesdale and Mr W.A. Ferguson who performed the routine biochemical assays.

### References

BRADFORD, M.M. (1976). A rapid and sensitive method for the quantitation of microgram quantities of protein utilizing the principle of protein-dye binding. *Analyt. Biochem.*, **72**, 248.

CANO, A., COFFER, A.I., ADATIA, R., MILLIS, R.R., RUBENS, R.D. & KING, R.J.B. (1986). Histochemical studies with an oestrogen receptor-related protein in human breast tumours. *Cancer Res.*, (in press).

COFFER, A.I. & KING, R.J.B. (1981). Antibodies to estradiol receptor from human myometrium. *J. Steroid Biochem.*, **14**, 1229.

COFFER, A.I., LEWIS, K.M., BROCKAS, A.J. & KING. R.J.C. (1985*a*). Monoclonal antibodies against a component related to soluble estrogen receptor. *Cancer Res.*, **45**, 3686.

COFFER, A.I., SPILLER, C.H., LEWIS, K.M. & KING, R.J.B. (1985*b*). Immunoradiometric studies with monoclonal antibody against a component related to human estrogen receptor. *Cancer Res.*, **45**, 3694.

COLLIN, P., NICHOLSON, R.I., FRANCIS, A.B., WILLIAMS, M., BLAMEY, R.B. & GRIFFITHS, K. (1985). Evaluation of an immunoradiometric assay for an oestrogen receptor associated antigen. *J. Endocrinol.*, **107**, Suppl. Abs. 54.

GREEN, S., WALTER, P., KUMAR, V. & 4 others (1986). Human oestrogen receptor c DNA: sequence, expression and homology to v-erb A. *Nature*, **320**, 134.

GREENE, G., FITCH, F.W. & JENSEN, E.V. (1980). Monoclonal antibodies to estrophilin: probes for the study of estrogen receptor. *Proc. Natl Acad. Sci. USA*, **77**, 157.

HAWKINS, R.A. (1985). Receptor assays in the management of breast cancer. *Brit. J. Hosp. Med.*, **34**, 160.

HAWKINS, R.A., BLACK, R., STEELE, R.J.C. DIXON, J.M.D. & FORREST, A.P.M. (1981). Oestrogen receptor concentration in primary breast cancer and axillary node metastases. *Breast Cancer Res. Treat.*, **1**, 245.

HAWKINS, R.A., ROBERTS, M.M. & FORREST, A.P.M. (1980). Oestrogen receptors and breast cancer: current status. *Br. J. Surg.*, **67**, 153.

HAWKINS, R.A., SANGSTER, K. & KRAJEWSKI, A. (1986). Histochemical detection of oestrogen receptors in breast carcinoma: a successful technique. *Br. J. Cancer*, **53**, 407.

JENSEN, E.V. & DE SOMBRE, E.R. (1973). Estrogen-receptor interaction. *Science*, **182**, 126.

KING, R.J.B., COFFER, A.I. & SPILLER, G. (1984). Immunoassay and immunohistochemical assay of oestradiol receptor in human tumours. *J. Endocrinol.*, **102**, Abstr. 64.

KING, R.J.B., COFFER, A.I., GILBERT, J. & 5 others. (1985). Histochemical studies with a monoclonal antibody-raised against – a partially purified soluble estradiol receptor preparation from human myometrium. *Cancer Res.*, **45**, 5728.

KING, W.J., DE SOMBRE, E.R., JENSEN, E.V. & GREENE, G.L. (1985). Comparison of immunocytochemical and steroid-binding assays for estrogen receptor in human breast tumours. *Cancer Res.*, **45**, 293.

LEE, S.H. (1978). Cytochemical study of oestrogen receptor in human mammary cancer. *Am. J. Clin. Path.*, **70**, 197.

LOPE-PIHIE, A., PATEL, M., KUSEL, J. & LEAKE, R.E. (1985). Quantification of oestrogen receptor by immunofluorescence. *Biochem. Soc. Trans.*, **13**, 178.

PERTSCHUK, L.P., EISENBERG, K.B., CARTER, A.C. & FELDMAN, J.G. (1985). Immunohistologic localisation of estrogen receptors in breast cancer with monoclonal antibodies. *Cancer*, **55**, 1513.

PERTSCHUK, L.P., GAETJENS, E., CARTER, A.C., BRIGATI, D.J., KIM, D.S. & FEALY, T.E. (1979). An improved histochemical method for the detection of oestrogen receptors in mammary cancer. *Am. J. Clin. Path.*, **71**, 504.

SCATCHARD, G. (1949). The attraction of proteins for small molecules and ions. *Ann. NY Acad. Sci.*, **51**, 660.

TAMURA, H., RAAM, J., SMEEDY, A. & PAPPAS, C.A. (1984). An update on the immunohistochemical localisation of estrogen receptors in mammary carcinomas utilizing polyclonal anti-receptor antibodies. *Eur. J. Cancer Clin. Oncol.*, **20**, 1261.

WALKER, R.A., COVE, D.H. & HOWELL, A. (1980). Histological detection of oestrogen receptors in human breast carcinomas. *Lancet*, **i**, 171.